# Evaluating Potential Cetacean Welfare Indicators from Video of Live Stranded Long-Finned Pilot Whales (*Globicephala melas edwardii*)

**DOI:** 10.3390/ani12141861

**Published:** 2022-07-21

**Authors:** Rebecca M. Boys, Ngaio J. Beausoleil, Matthew D. M. Pawley, Emma L. Betty, Karen A. Stockin

**Affiliations:** 1 Cetacean Ecology Research Group, School of Natural Sciences, College of Sciences, Massey University, Private Bag 102-904, Auckland 1142, New Zealand; e.l.betty@massey.ac.nz; 2 Animal Welfare Science and Bioethics Centre, School of Veterinary Science, College of Sciences, Massey University, Private Bag 11-222, Palmerston North 4442, New Zealand; n.j.beausoleil@massey.ac.nz; 3 School of Mathematical and Computational Sciences, College of Sciences, Massey University, Private Bag 102-904, Auckland 1142, New Zealand; m.pawley@massey.ac.nz

**Keywords:** animal welfare assessment, behaviour, human intervention, marine mammal, cetacean, management, stranding, wildlife

## Abstract

**Simple Summary:**

Cetacean strandings occur globally and can impact the welfare as well as the survival of the animals involved. Understanding the welfare status of stranded cetaceans is important to inform appropriate human intervention. However, there is a lack of knowledge on how to assess animal welfare in this context. Here, we used video footage of live stranded animals of four odontocete species to explore which proposed welfare indicators can be assessed at live stranding events. We identified and evaluated potential indicators that could be non-invasively assessed, including 10 non-behavioural and 2 composite behavioural indicators (category of many behaviours). The first data on the fine-scale behaviour of stranded odontocetes and associated human intervention during stranding responses are presented. Our findings suggest that remote assessments of stranded cetacean’s welfare states are feasible. These data provide the foundation to develop a systematic, structured welfare assessment framework specific to stranded cetaceans that can inform conservation and management decisions.

**Abstract:**

Despite the known benefit of considering welfare within wildlife conservation and management, there remains a lack of data to inform such evaluations. To assess animal welfare, relevant information must be captured scientifically and systematically. A key first step is identifying potential indicators of welfare and the practicality of their measurement. We assessed the feasibility of evaluating potential welfare indicators from opportunistically gathered video footage of four stranded odontocete species (*n* = 53) at 14 stranding events around New Zealand. The first stranded cetacean ethogram was compiled, including 30 different behaviours, 20 of which were observed in all four species. Additionally, thirteen types of human intervention were classified. A subset of 49 live stranded long-finned pilot whales (*Globicephala melas edwardii*) were assessed to determine indicator prevalence and to quantify behaviours. Four ‘welfare status’ and six ‘welfare alerting’ non-behavioural indicators could be consistently evaluated from the footage. Additionally, two composite behavioural indicators were feasible. Three human intervention types (present, watering, and touching) and five animal behaviours (tail flutter, dorsal fin flutter, head lift, tail lift, and head side-to-side) were prevalent (>40% of individuals). Our study highlights the potential for non-invasive, remote assessments via video footage and represents an initial step towards developing a systematic, holistic welfare assessment framework for stranded cetaceans.

## 1. Introduction

The welfare of free-ranging animals is increasingly recognised as important to conservation [1,2,3]. In addition, there is a growing acknowledgment that human activities may directly and indirectly compromise the welfare of wild animals [4]. However, the conservation of wildlife populations is often a focus of government regulations, policies, and biodiversity plans, and the welfare of the individual animals comprising such populations is often overlooked. This is despite the fact that animal survival, and thus conservation success, is inextricably linked to welfare [1,5].

Although the need to assess wild animal welfare has been highlighted [6,7,8,9], there are limited systematic, scientific protocols for such assessments [10]. Furthermore, detailed behavioural and physiological data from species in the wild are often lacking [11], hindering the development of welfare assessments for wild populations [12]. Thus, a first step to progressing systematic and holistic welfare assessment for free-living wild animals is developing methods to capture relevant data. Such data need to be species/taxon- and context-specific and should address known or suspected welfare concerns [12,13]. Furthermore, to provide information about the welfare state of the animal, science-based indicators that can be observed and/or measured must be identified [12,14,15,16].

In the context of free-swimming cetaceans, data on stress hormones [17,18,19], body condition [20,21,22], skin disease [23,24,25], and the impacts of anthropogenic activities on behaviour [26,27,28] have been collected. However, few studies interpret their findings in terms of welfare or discuss possible welfare implications [9]. During live strandings, cetaceans are subject to both natural [29,30] and anthropogenic stressors [31] that may affect their welfare and survival likelihood [32]. Unfortunately, thus far, such data concerning behavioural and physiological indicators have not been gathered.

Major knowledge gaps concerning the welfare of stranded cetaceans were identified by international experts to be interpreting behavioural and physiological parameters, diagnosing internal injuries, and making end-of-life decisions [32]. Furthermore, these experts stated that major barriers to assessing the welfare of stranded cetaceans related to the limited relevant data collection at strandings and the lack of experts available onsite to interpret parameters and assist in decision making [32]. Notably, the characterisation of stranded cetacean welfare by those experts aligned with contemporary animal welfare science, which interprets interrelated aspects of health, biological function, and behaviour in terms of their impact on the animal’s mental state [33,34]. Welfare assessments guided by such characterisations are often facilitated via the use of the Five Domains Model framework for assessing animal welfare [35]. In such a framework, the indicators in domains 1–4 are observed and/or measured, and their cumulative impacts are used to cautiously infer the animal’s potential affective state (mental state) in the fifth domain [5,36,37].

Subsequently, these same experts proposed a range of potential welfare indicators for stranded cetaceans [38]. The potential indicators could be grouped into three physical/functional (nutrition, physical environment, and health) and one situation-related domain (behavioural interactions) of the Five Domains Model [35]. The proposed indicators included animal-based parameters, reflecting some aspect of the physical (e.g., body condition), physiological (e.g., respiration rate), or behavioural state of the animal (e.g., vocalisation). Other indicators were resource-/management-based parameters, reflecting aspects of the stranded cetacean’s environment (e.g., substrate or duration stranded) or management (e.g., human interaction) that may influence its welfare [39,40]. Resource-/management-based indicators provide welfare-relevant information but do not provide direct evidence of the welfare state and are thus characterised as ‘welfare alerting’ [12]. Only animal-based indicators can provide direct information about the animal’s ‘welfare status’ and are often preferred in welfare assessments. However, some animal-based indicators may only be ‘welfare alerting’ in that they can indicate a predisposition for welfare impacts that relate to the animal itself rather than its environment, for example, an animal that is neonatal or unweaned. Welfare alerting indicators are generally more feasible and reliable to assess across time and different observers and are often non-invasive. They are therefore commonly applied in welfare assessments.

To successfully apply indicators in a welfare assessment framework, the feasibility of measuring the indicators, methods of measurement, and validity for inferring welfare states (i.e., mental states) from observable indicators must be evaluated [10,41,42]. In this study we examined the feasibility of assessing animal-based and resource-/management-based indicators proposed by experts at live cetacean stranding events [38]. Furthermore, since experts highlighted the need for assessments to be undertaken by remote, skilled personnel [32], we evaluated which indicators can be observed and/or measured using video footage gathered at live strandings.

Specifically, this study evaluates the use of video footage to (1) identify potential animal and resource-/management-based welfare indicators that could be feasibly measured, (2) examine why certain proposed welfare indicators cannot be identified and/or feasibly measured, and (3) assess whether indicators observed from pilot whales can be quantitatively evaluated via video. There are currently no ethograms available for stranded cetaceans, and there is limited detail on the types of human intervention employed at stranding events. Therefore, we sought to identify and characterise all stranded cetacean behaviours (animal-based indicators) displayed and to provide the first description of the types of human intervention (resource-/management-based indicators) that occurred at these same stranding events. Additionally, we examined whether the features of the stranding circumstances affected the prevalence, frequency, or duration of behaviours displayed by stranded pilot whales.

## 2. Materials and Methods

### 2.1. In-field Data Collection

Due to the stochastic nature of strandings, video footage was collected opportunistically at 14 live stranding events between August 2010 and March 2022 around New Zealand (Table S1). Filming occurred with 53 live stranded cetaceans involving four species of odontocete: long-finned pilot whale (*Globicephala melas edwardii*), pygmy killer whale (*Feresa attenuata*), Cuvier’s beaked whale (*Ziphus cavirostris*), and Gray’s beaked whale (*Mesoplodon grayi*). Most stranding events (11 events, 49 individuals) involved long-finned pilot whales, herein referred to as pilot whales. Accordingly, the analyses presented here focus only on pilot whales. Additional data from the other examined species are included in the initial ground-truthing only to identify and characterise all animal behavioural and human intervention indicators.

The camera set-up varied based on the opportunistic nature of events and equipment availability (Table S1). When the researchers could attend a stranding, two GoPro Hero 7 Black video cameras (GoPro Inc., San Mateo, CA, USA) were mounted on wooden stakes anchored into the ground at 1–2 m from the focal animal. Each camera was mounted at a height of 50–100 cm, positioned cranio-laterally, and angled caudally (0–45°) towards the tail flukes for each focal individual. These recordings were made at 720 p and 60 fps with a wide-angle view, allowing for the focal animal’s entire body to be observed bilaterally. Where researchers were unable to access the animals prior to re-floatation, footage was acquired from stranding personnel, including Department of Conservation (DOC) rangers (the government agency responsible for the management of stranding events), marine mammal medics, and the public using camera phones, GoPro cameras, or other similar video cameras. In such circumstances, the videographer stood 1–2 m from the animal and, when possible, positioned themselves cranio-laterally to the focal individual’s head, angling the camera caudally towards the tail flukes. The videographer alternated positions around the animal to enable the entire body to be observed bilaterally. In other cases, individuals were filmed from a lateral position, capturing the entire body on one side. The filming duration was dependent upon battery availability, time of day, and the stranding response procedures in progress.

### 2.2. Selection of Potential Welfare Indicators

Based on the opinions of an international panel of experts in cetacean biology, veterinary medicine, and/or animal welfare [38], we developed a list of theoretically observable/measurable parameters that could be used as potential welfare indicators for stranded cetaceans. The list also included parameters that were deemed observable/measurable from an initial viewing of the video footage collected during the stranding events (Table S1). The indicators and composite behavioural parameters (category including many different behaviours, each of which would be considered an indicator) were organised into the three physical/functional domains (nutrition, physical environment, and health) and the situation-related domain (behavioural interactions) of the Five Domains Model for welfare assessment [35] (Table 1). Within each domain, indicators were further split into animal-based indicators that may directly reflect animal state (‘welfare status’), and ‘welfare alerting’ indicators (both animal- and resource-/management-based), which provide relevant information about the animal or its environment that may affect its state [12] (Table 1).

### 2.3. Data Scoring

Each video file, for all species, was examined manually at 0.8× speed by the lead author (RMB) at least twice to identify all observable indicators for each focal individual. A subset of videos was examined by two independent observers to ensure consistency in indicator classification. For each animal, information was collated about which indicators could be assessed (Table 1) and, for those indicators that can vary bilaterally, whether they could only be assessed on the left, right, or both sides. The reasons that particular indicators could not be observed and/or measured for each individual cetacean were also noted. Since 92.5% (*n* = 49) of individual focal stranded cetaceans were pilot whales (Table S1), only data related to that species were subsequently analysed and are presented here. To be considered feasible, an indicator had to be fully assessable (across the whole body) and prevalent, being observed in at least 40% of the pilot whales.

**Domain 1: Nutrition** 

**Figure 1:**
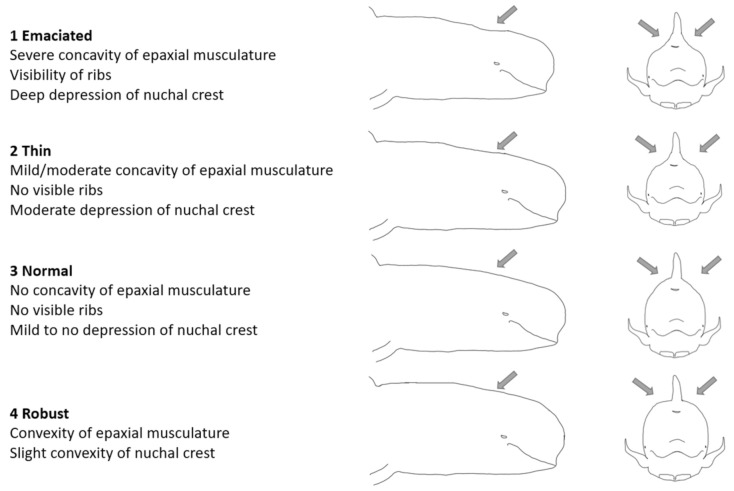
Four-point visual body condition scoring system developed for long-finned pilot whales (*Globicephala melas edwardii*) in this study.

Body condition was assessed visually based on the concavity of the epaxial musculature and nuchal crest following Joblon et al. [43]. The four-point body condition score was assessed as (1) emaciated: severe concavity of the epaxial musculature, visibility of the ribs, and deep depression of the nuchal crest; (2) thin: mild to moderate concavity of the epaxial musculature, no visible ribs, and moderate depression of the nuchal crest; (3) normal: no concavity of the epaxial musculature, no visible ribs, and mild to no depression of the nuchal crest; and (4) robust: convexity of the epaxial musculature and a slight convexity of the nuchal crest (Figure 1).

The age class of the animal was qualitatively assessed based on the approximate length relative to the known adult length for the species [44]. Animals were assigned to one of three categories: adult, juvenile, or calf. As the sex of all animals could not be assessed, we assigned adults to be those animals of more than ~432 cm [44]. Juveniles were estimated to be over one third of the length of an adult, while calves were determined to be less than one third of the adult length and/or with foetal folds still visible.

**Domain 2: Physical environment** 

**Figure 2:**
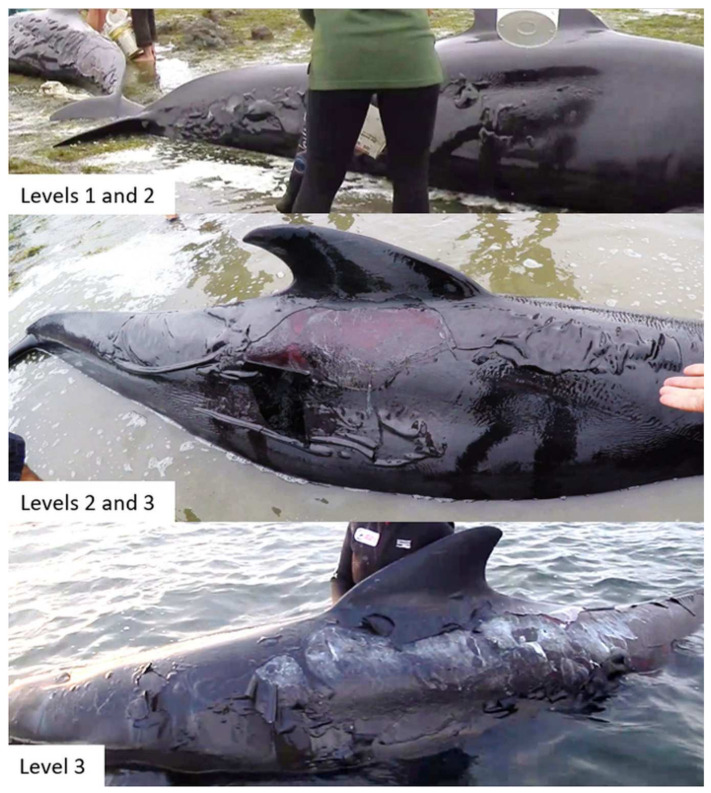
Level of skin blistering observed in individual focal animals. (**1**) Dermal necrosis and (**2**) bullae development on two individuals (top), (**2**) bullae development and (**3**) recent dermo-epidermal clefting with ulceration (middle), (**3**) dermo-epidermal clefting with ulceration two days after initial stranding (bottom). Photos credits: Kyle Mulinder (top and middle) and Project Jonah NZ (bottom).

The severity of any skin blistering was qualitatively scored following Groch et al. [45] based on the presence of superficial dermal necrosis (level 1), developed cutaneous bullae (level 2), or developed dermo-epidermal clefting with ulceration (level 3; Figure 2).

When available, information was collected from stranding response forms about the focal animal’s stranding circumstance, specifically, if this was an initial stranding or whether the animal had previously been re-floated and then subsequently re-stranded. Whether the animal was dry-stranded (i.e., on sand only, with no water around the whole body) or in-water-stranded (i.e., whole body surrounded by shallow water but not floating) was determined from the video footage. For animals that were filmed over a prolonged period, the animals were classified as dry-stranded or in-water-stranded based on the conditions present for the longest period during the filming.

The availability of basic stranding response equipment was assessed based on what was in view on the video footage of the focal individual. This included sheets for covering the animals, buckets for pouring water over the animal, spades for digging, and re-floatation mats. The substrate type was assessed from the video footage based on whether the focal animal was stranded on (1) mud flats, (2) sandy beach, (3) pebble beach, or (4) rocky shore. The substrate information was used to provide additional context to potential welfare concerns such as external injuries.

Weather and sea swell were assessed based on what could be viewed on the video. Weather conditions were categorised as (1) sunny, (2) overcast, or (3) precipitation. For animals filmed in prolonged stranding events, the weather conditions were classed as those most prevalent during filming. Due to the potential impact of swell height on the ability to attempt re-floatation, sea conditions were qualitatively assessed based on the approximate swell height as (1) minimal to small swell, ankle- to waist-high waves; (2) medium swell, waist- to shoulder-high waves, or (3) large swell, head-high and larger waves. The tidal conditions were assessed based on whether the tide was low or high and flooding or ebbing, based on tidal charts [46] for the specific stranding date, time, and location.

**Domain 3: Health** 

Externally visible injuries were qualitatively assessed as being superficial or penetrating wounds and were classified by the location on the body. Skin illness/disease was scored based on the perceived appearance of characterised cutaneous manifestations known to occur due to specific infections/diseases [23,24,47,48,49], including “tattoo”, “rounded cutaneous”, “whitish velvety”, and “whitish to slightly pink verrucous” skin lesions, following Van Bressem et al. [47]. Skin illness/disease lesions were assessed based on being present/absent and the area of the body involved [47].

The respiration rate was assessed based on the visible opening and closing of the blowhole and the audible sound of the focal animal exhaling. The respiration rate was quantitatively assessed, with each audible and visible open/close of the blowhole considered to be a single respiration [50]. The respiration character/effort was qualitatively assessed by examining whether the inhalation and closure of the blowhole occurred immediately following exhalation or there was a period (measured in seconds) between exhalation and inhalation [51,52]. Additionally, unusual respiration was noted through a qualitative assessment of the blowhole opening and closing and audible exhalation, such as whether the animal exhaled twice before inhaling or displayed chuffing [51,53].

The heart rate was quantified, when possible, as a recorded count of the rhythmic movement of the skin [54] on the ventrum, medial to the left pectoral fin. However, the heart rate was only observable in animals in lateral recumbency, as movement in the area close to the ventral surface of the left pectoral fin must be visible. Each of the animal’s observable orifices were examined throughout the video duration to assess for any blood, mucus, or other fluids being expelled. Any such excretions were noted qualitatively based on the frequency and the orifice of origin.

**Domain 4: Behavioural interactions** 

**Figure 3:**
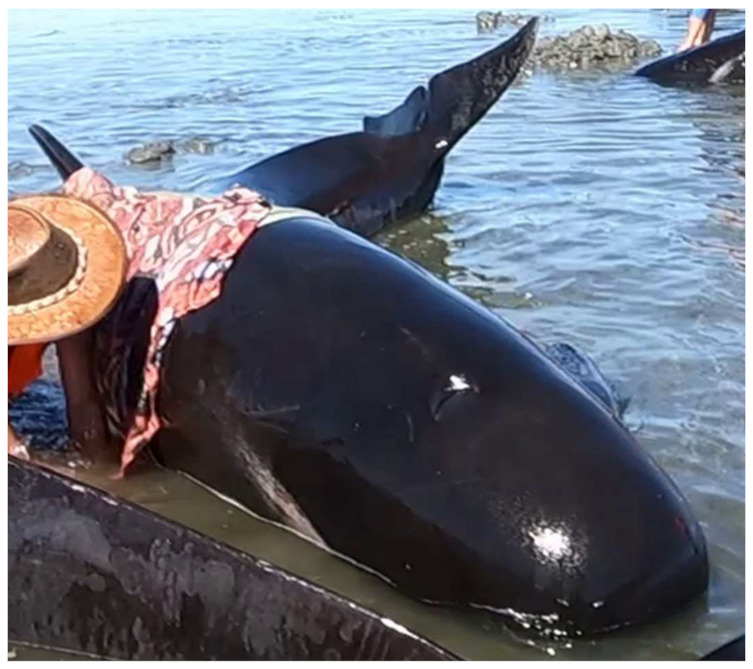
Example of spinal curvature: left lateral curvature of the peduncle in a stranded long-finned pilot whale. Photo credit: Kyle Mulinder.

Body posture was assessed based on the animal’s recumbency position: (1) ventral (lying on the ventrum), (2) lateral (lying on one side of the body), or (3) dorsal (lying on the dorsal surface of the body). Animals could be scored in multiple positions during a video; for example, they may have been moved from a lateral to a ventral position as part of the standard stranding response procedures [31]. Additionally, body posture was assessed based on whether the animal exhibited spinal curvature, most often observed in the peduncle. This feature was assessed based on continuous presence/absence throughout the observation period and was categorised as (1) lateral curvature: body or peduncle is curved laterally to the left or right (Figure 3), (2) dorsal curvature: peduncle is curved dorsally, or (3) ventral curvature: peduncle is curved ventrally.

Animal movements were assessed based on the type of behaviour and its prevalence, frequency, and relative duration (see Section 2.3.1). Additionally, audible vocalisations were assessed based on the presence/absence and duration as part of the behavioural analysis (see Section 2.3.1). Since it was not possible to confirm whether audible vocalisations were from the focal animal or another animal in the immediate vicinity during mass strandings, the social circumstances of the focal animal was noted when vocalisations were recorded.

Some video footage provided observation of additional stranded animals, and further information on the stranding event was gathered from DOC stranding reports. This enabled the evaluation of whether the pod members of the focal stranded animal were present/absent and, when present, whether pod members were (1) alive or dead and (2) stranded or floating. Human intervention was considered to occur when a human interacted with a focal animal (see Section 2.3.2). The intervention was assessed based on the presence/absence and type of interaction occurring.

#### 2.3.1. Development of Ethogram

Video footage was examined using the program BORIS v7.9.6 [55] to develop a comprehensive ethogram that represents all behaviours observed for the four species of stranded cetaceans, including unusual and rare occurrences [56]. The preliminary ethogram was based on five behaviours detailed in the literature relating to decision-making on the re-floatation of stranded odontocetes: body posture, arching, thrashing, trembling, and vocalisation [31,57,58,59]. However, these specific behaviours have not previously been described in detail, and their occurrence was not quantified in those studies. Thus, to begin, those behaviours were identified and defined for the two stranded pygmy killer whales due to the length of the video footage available (5 h; Table S1). The footage was then re-examined to characterise all other behaviours expressed by the pygmy killer whales until no new behaviours were noted. This updated ethogram (*n* = 20 behaviours) was then applied to the footage of the other species and stranding events, with new behaviours identified and characterised if there was no prior observation. Additionally, two physiological parameters (respiratory rate and heart rate) were included in the ethogram, as their frequency and duration could be calculated from video footage.

#### 2.3.2. Human Intervention

Video footage was also examined to identify and characterise the types of human intervention for inclusion in the ethogram. The footage for each focal cetacean was examined manually at 0.8× speed by the lead author (RMB) at least twice to identify and ensure intra-observer reliability of characterisation. Additionally, the same two independent observers examined a subset of videos to ensure consistency in the characterisation of intervention types.

Human intervention occurring at live stranding events includes up-righting animals, covering them in wet sheets, and pouring water over the body to reduce the risk of hyperthermia and sunburn [31]. However, previous studies have not provided a detailed characterisation of the types of human intervention occurring with live stranded cetaceans. In this study, a human intervention was considered to occur when a human was observed on the video footage within 1–2 metres of the focal cetacean. Again, the video footage of the pygmy killer whales was examined to characterise all types of human intervention until no new interventions were observed. This ethogram of human intervention was then applied to all other stranding events. New intervention types were identified and characterised if there was no prior observation.

### 2.4. Analysis of Pilot Whale Data

All behavioural and physiological parameters and human interventions identified in the ethogram for the 49 pilot whales were characterised and coded per second using BORIS v7.9.6 [55]. The prevalence, frequency, and relative duration of the behavioural and physiological parameters and human interventions were calculated from the quantitative scores and standardised by each video’s duration to remove any time bias. The behavioural parameters and human interventions were classified as point/event behaviours when they had very short and non-variable durations or as behavioural states when their durations varied. The prevalence of each behaviour and type of human intervention was determined as the percentage of individual pilot whales displaying the parameter or being exposed to the intervention at least once during the observation period. The frequency of point/event behaviours was calculated as the mean rate per minute, including only the individuals displaying that particular behaviour, and the variability was calculated as the standard error of the mean (SEM). The average relative duration of each state behaviour or human intervention was calculated as a percentage of the observation period, including only those individuals that displayed the behaviour or were exposed to the intervention, with variability presented as the range of relative durations.

We further examined whether the features of the stranding circumstances of the individual pilot whales affected the prevalence, frequency, or duration of expression of prevalent behavioural and physiological indicators. We examined the effect of stranding number (initial vs. re-strand) and circumstance (dry vs. in-water) on the prevalence of behaviours and physiological parameters using a Z-test for proportions and on the frequency of point/event behaviours and physiological parameters and the relative durations of state behaviours using a Mann–Whitney U test. To ensure valid statistical inferences, only prevalent parameters (observed in >40% individuals) were included in the analyses. The effects of these features on animals’ durations in different postural positions were not evaluated, as they were likely affected by human intervention rather than varying according to the focal animal’s state.

## 3. Results

A total of 427.2 min (7.1 h) of video footage was collected from 11 mass and 3 single stranding events, with observations of 53 focal individuals of four species (Table S1). The duration of focal individual observations ranged from 10 s to 212.9 min (3.6 h) (mean: 483.6 s; 8.1 min).

### 3.1. Feasibility of Welfare Indicators for Stranded Pilot Whales

There were 49 video clips of individual pilot whales, for a total of 93.5 min (1.6 h), with a mean length of 114.5 s (1.9 min). A total of 16 pilot whales (32.7%) were observable on both sides of the body, while 16 (32.7%) were observable on the left side only, and 17 (34.7%) were observable on the right side only. Table 2 shows the associated results of the 17 non-behavioural welfare indicators that were assessed. Of these, four welfare status indicators were feasible to assess from video footage of more than 40% of the stranded pilot whales. The welfare status indicators that could not be consistently assessed were heart rate, skin blistering, trauma/injuries, and skin disease. A further six welfare alerting indicators were also feasible to assess in at least 40% of pilot whales via video footage, while the other three indicators required data to be gathered from DOC stranding response forms.

**Domain 1: Nutrition** 

Body condition was feasible to fully assess in 29 (59.1%) stranded pilot whales. For the remaining animals, sheets covered the epaxial musculature (*n* = 20). Thus, the main visual assessment was based on the concavity of the nuchal crest. Most individuals (85.7%, *n* = 42) were in normal body condition and were adults (79.6%, *n* = 39).

**Domain 2: Physical environment** 

Due to animals being covered in sheets, just eight (16.3%) animals could be assessed across all body regions bilaterally. A further 21 (42.9%) could be assessed across all body regions on one side (10 on the left and 11 on the right). Of these, skin blistering was observed in 72.4% (*n* = 21). Skin blistering around the cranial region (including the mandibles, melon, and blowhole; see Figure 2) could be assessed in all animals—an additional seven animals had blistering present in this cranial region. The level of blistering varied among the 28 affected pilot whales (Table 2).

The stranding circumstance of being in-water- or dry-stranded was feasible to assess in 100% of cases (Table 2). Further information gathered from stranding reports indicated that, at the time of filming, more than half the animals had re-stranded.

The availability of basic stranding response equipment could be assessed in all cases, with variable equipment available (Table 2). The substrate at the stranding location was identified to be sandy in 100% of cases, though in three cases shells were present.

The weather was feasible to assess in all videos. For most animals (65.3%, *n* = 32), the weather was overcast, while for the remainder, it was sunny. Over half of the pilot whales were observed at low tide, with the tidal conditions varying for the rest. The distant low tide mark meant that the sea condition could not be assessed for 26 animals, while most of the remainder were observed with minimal swell (Table 2).

**Domain 3: Health** 

**Figure 4:**
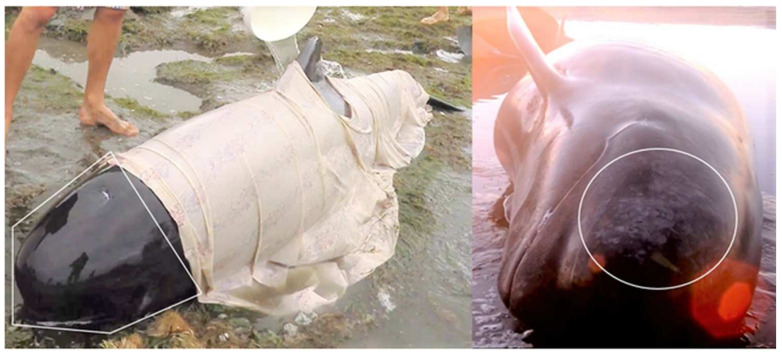
(**Left**): Observation of mucus from the blowhole and mouth of two live stranded long-finned pilot whales. Area considered the cranial region is defined within the white pentagon. (**Right**): Tattoo-like lesions (within white oval) observed on the cranial region of one individual. Photo credits: Kyle Mulinder (**Left**) and Project Jonah NZ (**Right**).

Injuries and wounds across the head and flukes were feasible to assess in all animals, while eight (16.3%) could be assessed across all body regions bilaterally, and 21 (42.9%) could be assessed on one side. Injuries and wounds were rare and mainly involved superficial lacerations (Table 2); these injuries in two animals were likely related to the substrate containing shells. Similarly, skin lesions indicative of illness/disease were not feasible to assess for the 20 animals covered by sheets, and a further 21 could only be assessed on one side. Of the 29 animals that were assessed, one was observed to have tattoo-like lesions on the cranial region (Figure 4; Table 2).

All respiratory events would have been observable via the video footage if they had occurred. However, due to the short length of some videos, respiration was only observed in 67.3% (*n* = 33) of the animals. In four of these animals, an unusual respiratory character was noted; one animal displayed double chuffing, with short forceful exhalations occurring twice prior to inhalation for almost every respiratory event. Three animals displayed an extended time between exhalation and inhalation. Indeed, in one animal the blowhole remained open for 6 s post-exhalation and prior to inhalation. The heart rate was only feasible to assess in three animals since the other individuals in the necessary position (lateral or dorsal recumbency) were in water (*n* = 9) or were filmed at an angle not conducive to observing the ventrum (*n* = 1).

Bleeding/fluid/mucus from orifices was readily assessable in the case of the blowhole and mouth (95.9%, *n* = 47) of the animals. Mucus excretion was observed to occur in four animals, two from the mouth and two from the blowhole (Figure 4). The genital and anal orifices were less observable due to most animals being in ventral recumbency (71.4%, *n* = 35). However, three animals were observed to defecate dark-green liquid (Figure S1).

**Domain 4: Behavioural interactions** 

Body posture was feasible to assess in 100% of the pilot whales, with most animals only in ventral or lateral recumbency throughout filming (Table 3). Nine (18.4%) were observed in both ventral and lateral recumbency, with an additional animal observed in ventral, lateral, and dorsal recumbency over 1.5 min. Spinal curvature (Figure 3) was feasible to assess in all cetaceans and was noted in ten individuals (20.4%; Table 2). Notably, four pilot whales had their pectoral fins oriented laterally and superior to the dorsal plane (Figure S2), and all were undergoing human intervention when filmed. Behavioural events were observed in 100% of individuals; detailed results are presented in Section 3.2. Audible vocalisations from animals were only detected at mass strandings; these were identified from video of five focal animals (10.2%; Table 3), three of which were identified as calves and two were adults in the presence of calves.

Nearly all (95.9%, *n* = 47) of the focal individuals formed part of mass strandings and, therefore, stranded conspecifics were also present (Table 2). Human interactions with focal animals were observed in 100% of events and included non-invasive (presence only) and invasive interactions (e.g., up-righting animals). Detailed information on the observed human interactions is provided in Section 3.3.

### 3.2. The Stranded Odontocete Ethogram

Thirty behaviours were identified and described for the four odontocete species when stranded. These included 6 point and 24 state behaviours (Table S2). Aside from the recumbency posture, behavioural parameters were not mutually exclusive in that multiple behaviours could be displayed by an individual simultaneously.

#### 3.2.1. Quantifying Behavioural Observations: Pilot Whales

Table 3 shows how feasible each of the behavioural indicators were and provides the prevalence, frequency, and duration of the assessed behavioural and physiological parameters. Notably, almost all the behavioural indicators (93.3%, *n* = 28) would have been feasible to assess if they had occurred. Eye open left and right were not consistently feasible due to light conditions and pec joint movement was not feasible in covered animals.

Most pilot whales (71.4%, *n* = 35) were observed in ventral recumbency throughout filming. When this recumbency was noted, it lasted for an average of 88.5% of the observation period. A further 10 individuals were moved into ventral recumbency as part of the human intervention during filming. The remaining individuals were filmed in lateral recumbency (28.6%, *n* = 14), which on average, lasted for 60.7% of the observation.

Five behaviours were prevalent, being displayed by over 40% of the pilot whales: tail flutter (69.4%, *n* = 34), dorsal fin flutter (55.1%, *n* = 27), head lift (51%, *n* = 25), tail lift (46.9%, *n* = 23), and head side-to-side (42.9%, *n* = 21). The only behaviour observed in other species but not recorded in pilot whales was head arch. In contrast, nine behaviours were recorded only in pilot whales (Table S2), all with low prevalence (Table 3).

When observed, individuals spent, on average, more than half the monitored time displaying right pectoral fin flutter (57.7%) and tail flutter (54.6%). The mean percentage of the observation period spent displaying dorsal fin flutter in those that did was 41.4%. Although prevalent, head lifting occurred, on average, for only 12.3% of the observation period, whereas tail lift and head side-to-side, also both prevalent, occurred for nearly a quarter of the observation period. All point behaviours had low prevalence and rates of occurrence.

Respiration was recorded at a mean rate of 4.4 breaths/min (SEM ± 0.4). Notably, inspiration occurred simultaneously with head lifting in nearly 45% of occurrences. The mean recorded heart rate was 48.8 beats/min (SEM ± 11.6).

**Table 3:** Observed prevalence (% of individuals displaying or for which the indicator was feasible), mean frequency (rate/minute), or mean relative duration (% of observation period and range) for only long-finned pilot whales that displayed the behaviour, from a total of 49 individuals across 11 stranding events between August 2010 and March 2022. See Table S2 for descriptions of behaviours.

Behaviour	Prevalence	Frequency	Relative Duration
**State behaviours**			
Ventral recumbency	91.8		88.5 (7.7–100.0)
Lateral recumbency	28.6		60.7 (4.4–99.8)
Dorsal recumbency	2.0		3.4 (3.4–3.4)
Tail flutter	69.4		54.6 (4.6–99.9)
Dorsal fin flutter	55.1		41.4 (6.1–97.3)
Head lift	51.0		12.3 (2.3–32.6)
Tail lift	46.9		23.9 (0.6–72.9)
Head side-to-side	42.9		22.2 (1.1–81.5)
Pec fin flutter R	24.5		57.7 (3.8–98.6)
Pec fin flutter L	22.4		34.8 (4.3–34.75)
Pec joint moves	20.4		22.5 (0.5–78.3)
Tail hover	18.4		22.0 (0.2–55.2)
Tail side-to-side	16.3		15.8 (0.3–46.3)
Body tremble	12.2		24.3 (0.2–84.2)
Vocalisation	10.2		20.7 (5.1–60.2)
Body rocking	10.2		10.4 (5.2–23.2)
Eye open L	10.2		35.4 (2.4–73.7)
Eye open R	8.2		22.1 (2.5–63.8)
Body tenses	6.1		11.2 (5.1–20)
Tail arch	4.1		15.8 (12.4–19.1)
Tail fluke slapping	4.1		28.3 (19.9–36.7)
Whole body arching/thrashing	4.1		5.4 (2.4–8.4)
Mouth open	2.0		17.2 (17.2–17.2)
**Point behaviours**			
Blowhole twitch	22.4	4.7 ± 1.7	
Nuchal pad twitch	10.2	6.2 ± 2.9	
Open and close blowhole	6.1	3.0 ± 1.6	
Water from blowhole	4.1	2.1 ± 1.4	
Head–pec fin jerk/flinch	2.0	2.6 ± 0.0	
Movement in lower jaw	2.0	6.9 ± 0.0	
**Physiological parameters**			
Respiration	67.3	4.4 ± 0.4	
Heartbeat	6.1	48.8 ± 11.6	

##### Differences in Stranding Circumstances: Initial vs. Re-Strand

Of the 49 pilot whales observed, 29 (59.2%) were filmed during a re-stranding, while the remainder (40.8%, *n* = 20) were filmed during their initial stranding event. Body tremble, mouth open, and movement in the lower jaw were only displayed by animals that were stranded for the first time, while the head–pec fin jerk was only observed in re-stranded animals (Table 4).

One prevalent behaviour, dorsal fin flutter, was displayed by a significantly (z = 2.33, *p* = 0.03) greater proportion of initially stranded animals than re-stranded animals (Table 4). No evidence of differences in the duration of prevalent behaviours or the rate of respiration was detected (z = −0.83, *p* = 0.41; Table 4).

**Table 4:** Observed prevalence (% of individuals displaying behaviour), mean frequency (rate/minute) ± SEM of point behaviours, and mean relative duration (% of monitored time and range) of state behaviours for only long-finned pilot whales that showed the behaviour, from a total of 20 initial-stranded and 29 re-stranded individuals across 11 stranding events on the New Zealand coast between 2010 and March 2022. ^†^ Only prevalent indicators could be tested for statistical differences; * significant difference (*α* = 0.05) in prevalence between stranding circumstances.

	Prevalence		Frequency		Relative Duration	
Behaviour	Initial Strand	Re-Strand	Initial Strand	Re-Strand	Initial Strand	Re-Strand
**State behaviours**						
Ventral recumbency	90.0	93.1			85.9 (17.5–100.0)	90.3 (7.7–99.9)
Lateral recumbency	40.0	20.7			54.9 (4.4–99.8)	68.4 (7.2–97.4)
Dorsal recumbency	0.0	3.4			0.0 (0.0–0.0)	3.4 (3.4–3.4)
Tail flutter ^†^	75.0	65.5			49.3 (4.6–99.9)	58.8 (12.9–98.1)
Dorsal fin flutter^†^	75.0 *	41.4 *			38.0 (6.1–95.6)	45.6 (13.0–97.3)
Head lift ^†^	65.0	41.4			12.2 (2.6–32.4)	12.4 (2.3–32.6)
Tail lift ^†^	45.0	48.3			27.4 (0.6–72.9)	21.6 (2.3–54.1)
Head side-to-side ^†^	60.0	31.0			22.6 (5.5–81.5)	21.7 (1.1–47.4)
Pec fin flutter R	35.0	17.2			55.2 (3.8–98.6)	61.2 (30.0–93.7)
Pec fin flutter L	30.0	17.2			22.7 (4.3–69.2)	49.2 (12.2–87.0)
Pec joint moves	25.0	17.2			10.6 (0.5–30.0)	34.5 (11.3–78.3)
Tail hover	20.0	17.2			20.0 (0.2–55.2)	23.6 (2.2–55.1)
Tail side-to-side	15.0	17.2			31.1 (11.7–46.3)	6.7 (0.3–11.8)
Body tremble	30.0	0.0			24.3 (0.2–84.2)	0.0 (0.0–0.0)
Vocalisation	10.0	10.3			33.5 (6.7–60.2)	12.2 (5.1–24.4)
Body rocking	15.0	6.9			11.7 (5.2–23.2)	8.6 (5.9–11.3)
Eye open L	15.0	6.9			37.5 (14.2–73.7)	32.2 (2.4–61.9)
Eye open R	10.0	6.9			33.2 (2.5–63.8)	11.1 (2.9–19.3)
Body tenses	10.0	3.4			14.2 (8.4–20.0)	5.1 (5.1–5.1)
Tail arch	5.0	3.4			19.1 (19.1–19.1)	12.4 (12.4–12.4)
Tail fluke slapping	5.0	3.4			19.9 (19.9–19.9)	36.7 (36.7–36.7)
Whole body arching/thrashing	5.0	3.4			8.4 (8.4–8.4)	2.4 (2.4–2.4)
Mouth open	5.0	0.0			17.2 (17.2–17.2)	0.0 (0.0–0.0)
**Point behaviours**						
Blowhole twitch	25.0	20.7	6.2 ± 3.0	5.3 ± 1.9		
Nuchal pad twitch	10.0	10.3	1.9 ± 0.7	9.6 ± 4.0		
Open and close blowhole	10.0	3.4	2.8 ± 1.2	5.9 ± 0.0		
Water from blowhole	5.0	3.4	3.6 ± 0.0	0.7 ± 0.0		
Head–pec fin jerk/flinch	0.0	3.4	0.0 ± 0.0	2.6 ± 0.0		
Movement in lower jaw	5.0	0.0	6.9 ± 0.0	0.0 ± 0.0		
**Physiological parameters**						
Respiration rate ^†^	65.0	69.0	3.8 ± 0.7	4.6 ± 0.6		
Heartbeat	10.0	3.4	33.8 and 71.5	41.1		

##### Differences in Circumstance: Dry vs. In-Water Strandings

Eighteen pilot whales (36.7%) were observed stranded in water, while 31 (63.3%) were recorded as dry-stranded. Body rocking and tail fluke slapping were only observed in individuals that were dry-stranded, while tail side-to-side, tail arch, whole body arching/thrashing, mouth open, head–pec fin jerk, and movement in the lower jaw were only displayed by animals stranded in water (Table 5).

Four prevalent behaviours were displayed in a significantly greater proportion of animals stranded in-water than dry-stranded animals: dorsal fin flutter (z = −3.03, *p* = 0.00), head lift (z = −2.26, *p* = 0.03), tail lift (z = −3.29, *p* = 0.00), and head side-to-side (z = −2.57, *p* = 0.02; Table 5). However, no evidence of differences was observed in the duration of prevalent behaviours or in the rate of respiration (z = −0.97, *p* = 0.33; Table 5).

### 3.3. Human Intervention with Stranded Odontocetes

From video footage of all stranded odontocetes, a total of 1061 events were coded from 13 different human interventions (Table S3). The types of human intervention were not mutually exclusive. Indeed, some types of human intervention always occurred simultaneously (e.g., human rolling an individual also required direct contact with the stranded animal).

#### Quantifying Human Intervention with Stranded Pilot Whales

All types of human intervention would have been feasible to assess if they occurred with the stranded pilot whales. Humans were present at all pilot whale stranding events that were observed, and on average, a human was within 2 metres of the focal animal (present) for 97% of the observed time (Table 6). Aside from human presence, the interventions that were most prevalent, occurring with over half of the stranded pilot whales, were human watering (65%) and human touching (59%). The interactions with the longest average duration per individual focal animal, aside from human presence, were human places sand by sides (96.8%), human touching (61.1%), and human noise (61.2%; Table 6).

## 4. Discussion

A range of potential animal- and resource-/management-based welfare indicators were able to be non-invasively observed and/or measured in stranded cetaceans. We systematically characterised, for the first time, the ethology of stranded odontocetes with 30 different behaviours described. We quantitatively assessed these welfare indicators, including fine-scale behaviour and human intervention, from 49 live stranded pilot whales. Previous studies have highlighted the need for systematic assessment of wild cetacean welfare but have also emphasised challenges due to limited behavioural and physiological data [60,61]. Our study contributes pivotal baseline data that can be used to develop a feasible welfare assessment framework specific to cetacean strandings.

### 4.1. Holistic Welfare Assessments Are Feasible at Cetacean Stranding Events

A range of indicators related to different aspects of welfare were feasibly evaluated via video footage captured at cetacean strandings. Not only is this useful to enable remote experts to undertake animal assessments [32,38] but the non-invasive measurability of these indicators minimises further welfare compromise for cetaceans that are experiencing physiological stress [29,30]. Although invasive measures (e.g., blood sampling to evaluate haematological parameters) are informative for assessing the health of wild cetaceans [62,63,64], the use of non-invasive methods for welfare assessments is preferable. Further focus should be to validate the scoring of these indicators from video against live observations and among various indicators that reflect the health and welfare status as well as with known survivorship data.

From the 18 proposed indicators and composite behavioural parameters (Table 1), 10 non-behavioural, 5 animal behaviour, and 3 human intervention indicators were prevalent and thus were feasible to assess from video footage. Importantly, the identified feasible indicators were representative of three physical/functional domains (nutrition, physical environment, and health) and one situation-related domain (behavioural interactions) of the Five Domains Model [35], suggesting that holistic welfare assessments of stranded cetaceans could be achievable using these indicators. Of these, nine welfare status indicators represented three domains. The most feasible to assess were body condition (D1: nutrition), respiration and bleeding/fluid/mucus from orifices (D3: health), and body posture and composite behavioural indicators (D4: behavioural interactions). Potential welfare alerting indicators that could be consistently assessed were the age class (D1), substrate type (D2), dry vs. in-water stranding (D2), the availability of equipment and weather conditions (D2), the presence of other pod members (D4), and the composite behavioural indicator related to the amount and type of human intervention (D4).

Some potential indicators could not be consistently assessed from the video footage. Heart rate could not be evaluated in most animals, as this required a postural position of lateral or dorsal recumbency. However, we do not recommend that stranded cetaceans be placed into lateral recumbency to facilitate the assessment of heart rate, as this may cause pulmonary compression [31]. Thus, heart rate is unlikely to be feasible as a remotely assessed indicator of the welfare state of stranded cetaceans, though in-field assessments via palpation may be possible with trained personnel.

Trauma/injuries, skin blistering, and skin disease could not be assessed across all body regions bilaterally in about 40% of pilot whales, as they were covered to reduce hyperthermia and sunburn risk [31]. Furthermore, in more than two thirds of cases, bilateral observation of an animal’s body was not possible due to camera positioning. These factors likely negatively biased the prevalence of observed blistering and injuries. However, if systematic assessment frameworks were implemented to guide evaluations at strandings, video and/or photographs of all body regions could be rapidly captured before interventions occur, allowing for a subsequent assessment of these indicators. This would require minimal time involvement and thus would be unlikely to cause additional welfare compromise. The application of such a framework would ensure consideration of all relevant welfare information and facilitate holistic, multidimensional assessments [5,65].

Additionally, although respiratory events were feasible to assess in all video footage if they occurred, the short duration of some videos utilized in this study compromised our ability to assess the respiratory rate for every individual. Importantly, cetacean species have extended breath holds [66]. Thus, video footage should be collected for at least 5 min to enable assessment of the respiratory rate.

Our results suggest a similar behavioural repertoire among stranded odontocete species. Only one behaviour was not displayed by pilot whales (head arch), and this was only exhibited by two animals, one pygmy killer whale and one Cuvier’s beaked whale, possibly indicating severe physiological stress [57,67]. In contrast, nine behaviours were only displayed by pilot whales, likely due to the small sample size of the other species (*n* = 4). Therefore, our findings contribute valuable baseline ethological data from which other studies can assess stranded odontocete behaviour, though future efforts should further examine species-specific differences.

Information on environmental conditions is important to provide context when interpreting welfare status indicators, such as behaviours, and can influence management decisions [42]. In our study, the substrate, whether animals were dry- or in-water-stranded, and the weather conditions could be easily assessed from video footage. However, other alerting indicators required additional information, for example, determining whether individuals were re-stranded required access to stranding reports. Multiple stranding events can cause compounding damage and sustained stress [30], which likely compromise both welfare and survival likelihood [30,32].

Interestingly, almost 60% of the pilot whales had stranded more than once when observed, suggesting that re-floated animals often do not remain at sea, despite re-floatation being considered a ‘success’ [68]. We examined whether stranding circumstances (re-stranded vs. initially stranded and dry- vs. in-water-stranded) affected the prevalent behaviours displayed by pilot whales, with some differences found (See Section 4.2 for further discussion). However, further data collection is required to enable correlations among resource-/management-based indicators and animal-based indicators to better understand the welfare risk they reflect [69].

### 4.2. Preliminary Welfare Assessment of Stranded Pilot Whales

Most pilot whales observed were mass stranded and were assessed as adults in normal body condition based on an external visual assessment of the epaxial musculature and the concavity of the nuchal crest [43]. This outwardly healthy appearance has been reported previously at mass strandings [70,71] and generally suggests that hunger or sickness likely have minimal impacts upon these individuals. In contrast, two single stranded animals were in poor (thin) body condition, suggesting they were likely experiencing welfare compromise in the form of hunger and thirst prior to stranding. Indeed, one of these individuals was a neonate that likely stranded due to maternal–filial separation [72,73], suggesting that the welfare of this animal was significantly compromised at stranding. Such animals are also suggested to have low survival likelihood, and end-of-life decisions or long-term captivity are generally indicated [74,75].

Few injuries were observed, with those noted considered to be superficial. These may have occurred due to the stranding event itself and were likely minimal due to the sandy substrate. External injuries are less frequently observed in mass stranded animals, whereas single strandings can be related to some form of trauma [72,76]. Likewise, fluid or mucus discharge from the mouth or blowhole was rare and, when present, was mild. Additionally, faeces were evident from only three animals involved in the same mass stranding. The presence of vomiting and/or faecal discharges can be indicative of underlying health conditions [77] as well as indicating that animals are stressed [57,58]. Prolonged vomiting or diarrhoea can lead to dehydration and therefore should be considered welfare-relevant and included in evaluations.

Notably, despite widespread human interventions, such as covering, and overcast weather conditions, nearly 60% of animals had skin blistering, with serious blistering developed on more than a third. Both the number of affected animals and the severity of skin blistering were likely underestimated since most individuals were covered in sheets and/or had only one side of the body visible in the videos. The common occurrence corroborates the opinions of experts who indicated sunburn as a major welfare concern [32] and suggested it as an indicator for assessing stranded cetacean welfare [38]. Severe forms involving dermo-epidermal clefting with ulceration (observed in 20.4% of pilot whales) are likely to cause pain [38] and critical fluid loss [78], leading to dehydration, hypovolemic shock [45,79], and potential infection. Our results suggest there is considerable cause for welfare concern for many ‘managed’ live stranded pilot whales based on this indicator alone. Additional assessment of weather conditions will be useful to predict any further skin damage that may occur. Future studies should assess the extent of fluid, protein, and electrolyte loss that may occur when bullae ulcerate and rupture, as this will likely impact both the welfare and survivorship of stranded cetaceans. We suggest that such indicator data will also be important to inform decision making around re-floatation versus euthanasia.

Lateral curvature of the caudal peduncle was noted in 20% of animals in all stranding circumstances. This posture has been reported in stranded cetaceans during rehabilitation and is proposed to predict reduced swimming ability and muscular myopathy [80,81]. Additionally, four animals were observed with their pectoral fins oriented laterally and superior to the dorsal plane, which may indicate damage to joints, such as dislocations. Such postural abnormalities and/or underlying muscle or joint damage are likely to cause pain and, in the longer term, may detrimentally affect swimming and foraging ability [82]. Thus, such individuals may be deemed non-releasable [57,75,81,83]. Postural abnormalities should be correlated with other behavioural, physiological, and/or pathological indicators to better understand their welfare significance [42] and inform the use of this indicator in welfare assessments [13].

Almost all animals were observed in ventral recumbency for most of the video footage. This is likely due to the fact that human intervention occurred at all stranding events, and righting stranded cetaceans onto their ventrum is part of standard stranding response procedures [31,84]. This recumbency position is thought to reduce pulmonary compression compared to lateral recumbency [31] and should minimise the discomfort associated with breathing. Therefore, recumbency position should be considered in welfare assessments.

Interestingly, vocalisation during filming was rare and was only heard where focal animals were calves or adults in the presence of a calf, suggesting a possible maternal–filial connection. Previous studies suggest vocalisations are linked to cetacean welfare state in captive situations [85,86,87] and may affect epimeletic behaviour provided to wild distressed conspecifics [88]. Accordingly, we recommend additional data collection at stranding events to further assess the validity of vocalisations as a welfare indicator and to compile a comprehensive vocal repertoire for strandings.

All point behaviours had low prevalence and low rates of occurrence, meaning they will not be useful parameters for detecting any effects of environmental conditions or human interventions on cetacean welfare. In contrast, five state behaviours were prevalent, being displayed by more than 40% of the pilot whales (tail flutter, dorsal fin flutter, head lift, tail lift, and head side-to-side). When expressed, tail flutter and dorsal fin flutter were displayed, on average, for more than 40% of the observation time. Additionally, though less prevalent, right pectoral fin flutter occurred for more than 50% of observation time when expressed. Fin fluttering behaviours may be forms of muscle fasciculations or tremors. These fasciculations have previously been suggested as clinical signs of capture myopathy [30,81] and underlying health conditions [89]. Therefore, they are important to consider in welfare assessments.

Notably, dorsal fin flutter was observed in a significantly higher proportion of initial-stranded animals than re-stranded animals and in a greater proportion of in-water-strandings than dry-strandings. In the case of initial versus re-stranded animals, it may be that re-stranded animals become too fatigued to display dorsal fin fluttering. However, in the case of in-water versus dry-stranding, the expression of the behaviour appears to be context-specific and thus may represent the animal’s response to its situation. Therefore, the use of such a behaviour as a welfare indicator must consider the animal’s conditions and must be interpreted in the specific context of the stranding. Such behaviours may also be affected by human interventions and thus could be used to evaluate the effects on potential welfare state [90]. Future work should correlate these behaviours with physiological and/or pathological indicators to validate their reflection of welfare states [13] and inform their use in decision making.

Although prevalent within the study population, head and tail lift were displayed on average for only 12% and 24% of the observation time, respectively. Notably, both behaviours occurred in a significantly larger proportion of animals that were in-water-stranded than dry-stranded, suggesting that their expression may be context-specific. However, these behaviours may be precursors to arching, which was not observed in pilot whales but is proposed to be a sign of severe physiological stress in cetaceans [57,67]. Further data collection on these behaviours and their correlations with the specific stranding contexts should be undertaken to better understand the welfare state they may reflect and inform their use in welfare assessments for decision-making.

Many of the head lifting events occurred simultaneously with respiration. This is likely due to compression of the thoracic cavity when the animal is not supported by water (the case for all pilot whales in this study), which can cause breathing difficulties [51,67]. Furthermore, three pilot whales from the same mass stranding displayed delayed inhalation following exhalation for up to 6 seconds. Such respiratory delays are suggested to be indicative of shock and typically imply an end-of-life decision [51]. Further observation of head lifting during respiration events and delayed inhalation and the correlation of these behaviours with pathology will be important to assess, as this could provide data to infer the unpleasant experience of breathlessness [91,92]. These indicators should be considered important aspects to include in welfare assessments [69,93] and to inform decision-making around re-floatation versus euthanasia.

There were negligible differences in the frequency and duration of prevalent behaviours between initial-stranded animals and those observed during re-stranding and between dry- and in-water-stranded animals. This may be due to the inherent physiologically stressful situation of stranding, whereby behavioural differences caused by stranding circumstances are likely minimal. However, it is also possible that the lack of statistically significant effects is due to the sample size being too small to detect biologically relevant differences in behavioural expression. These common behaviours should be further correlated with physiological and/or pathological indicators to better understand their welfare significance [13]. They can then be considered for investigating the effects of various human interventions or stranding situations on animal welfare [42,90,93].

Human presence occurred nearly constantly for almost all observed pilot whales. Watering, touching, and digging out occurred with more than a third of the pilot whales and, when occurring, lasted for more than a third of the observation period. These high levels of interventions may negatively affect the welfare state of stranded cetaceans since humans may be perceived as threatening [35], particularly when encountered in an inherently physiologically stressful situation. However, appropriate, minimal intervention may also reduce other welfare concerns. For example, the provision of sheets and cooling water over the body should reduce the risk of hyperthermia and sunburn [31], which may otherwise cause pain and discomfort [32,38]. Future research should examine differences in stranded cetacean behavioural and physiological parameters with and without human intervention to investigate the effects of differing interventions on animal welfare [13].

### 4.3. Study Considerations

Due to the stochastic nature of stranding events, opportunistic filming by the public was an important data source in our study. Despite many videos being short in duration, we were able to identify and evaluate physical and environmental indicators and characterise behaviour. Similar video lengths have been used elsewhere [10,94]. However, these data are unlikely to provide accurate estimates of the behavioural time budgets and respiratory rates of stranded cetaceans. Furthermore, welfare compromise is expected to worsen throughout a stranding [30,32], and time stranded is considered a major concern for survival likelihood [32]. Accordingly, we recommend the application of standardized methods for data collection as a routine part of cetacean stranding response. This should include video recording from cameras mounted on poles and a longer filming duration, ideally from the onset of stranding to re-floatation or euthanasia, in order to fully evaluate the severity, duration, and progression of welfare impacts [95,96]. Standardized and continuous automated data collection will facilitate further investigation of indicators and the effects of human activities without hindering timely intervention to improve animal welfare and survival likelihood.

The experts consulted in Boys et al. [38], considered animal responsiveness via reflex testing to be a valuable and practical indicator. However, this was not tested at the stranding events presented here, despite it featuring in the New Zealand Standard Operating Procedures for cetacean strandings [75]. Nonetheless, it is likely that responsiveness could be evaluated via video footage with correct camera positioning. Thus, its feasibility should be assessed at future stranding events. Other valuable measures, such as body temperature, may also be taken in-field to augment the remote evaluation from video, though this may be limited by equipment and the availability of appropriately trained/skilled personnel. Finally, future studies should aim to collect data from both single and mass strandings to enable the statistical evaluation of the effects of stranding type on the presented indicators. The evaluation of these additional data will ensure comprehensive welfare assessments at future stranding events to inform decision making.

## 5. Conclusions

Video data provided valuable welfare-relevant information and highlighted the potential for experts to undertake assessments remotely. Importantly, our findings present an initial proof of concept concerning the feasibility of non-invasive welfare indicators, including behaviour, relevant to stranded odontocetes. However, additional data are required to explore the value of such indicators for predicting stranding outcomes, such as remaining at sea following re-floatation and longer-term survival, and to understand the effects of environmental conditions and human interventions on welfare and survivorship. Such information will better support decision-making concerning re-floatation versus euthanasia. Our study highlights the value of applying the Five Domains Model to facilitate holistic welfare assessments, allowing for more rapid informed prognoses of individual cetaceans. Including indicators that are practical to measure and validated in welfare assessment protocols will allow for more holistic, transparent, and justifiable evaluations of stranded cetacean welfare states. This will facilitate appropriate management interventions, leading to the best animal welfare and conservation outcomes from stranding events.

## Figures and Tables

**Table 1:** Proposed animal welfare indicators, or composite indicators *, organised into the three physical/functional domains and one situation-related domain of the Five Domains Model for welfare assessment [35]. Within each domain, indicators are organised according to the type of information they provide about the animal’s state. See text for details of each indicator and how it was measured or scored.

Domain	Indicators	
	Welfare Status	Welfare Alerting
1: Nutrition	Body condition	Animal age class
2: Physical environment	Skin condition/blistering	Initial strand vs. re-strand Dry strand vs. in-water strand Availability of equipment Substrate type * Weather, sea, and tidal conditions
3: Health	Signs of trauma, injuries Signs of skin illness and disease Respiration rate and character/effort Heart rate Bleeding/fluids/mucus from orifices	
4: Behavioural interactions	Body posture * Movements and behaviours Animal vocalisation	Presence and status of pod members * Type and duration of human interaction

**Table 2:** Non-behavioural welfare indicators assessed for 49 live stranded long-finned pilot whales across 11 stranding events between August 2010 and March 2022 on the New Zealand coast. The number of animals for which the indicator was feasible to assess across the whole body and the percentage of animals for each parameter.

Domain	Welfare Status Indicator (no. Feasible)	Percentage of 49 Individuals (n)	Welfare Alerting Indicator (no. Feasible)	Percentage of 49 Individuals (n)
1: Nutrition				
	**Body condition (29)**		**Animal age class (49)**	
	Thin	14.3 (7)	Adult	79.6 (39)
	Normal	85.7 (42)	Juvenile	10.2 (5)
			Calf	10.2 (5)
2: Physical environment				
	**Skin blistering (8)**		**Substrate type (49)**	
	Superficial dermal necrosis	18.4 (9)	Sand beach	100 (49)
	Cutaneous bullae	18.4 (9)	**Stranding circumstance (49)**	
	Dermo-epidermal clefting/ulceration	20.4 (10)	Initial strand	40.8 (20)
			Re-strand	59.2 (29)
			Dry strand	63.3 (31)
			In-water strand	36.7 (18)
			**Equipment (49)**	
			Sheets covering animal	40.8 (20)
			Buckets pouring water	65.3 (32)
			Spades	20.4 (10)
			**Weather (49)**	
			Sun	34.7 (17)
			Overcast	65.3 (32)
			**Sea condition (24)**	
			Minimal/small swell	44.9 (22)
			Medium swell	2.0 (1)
			**Tide (49)**	
			High	20.4 (10)
			Low	55 (27)
			Incoming	22.4 (11)
			Receding	2.0 (1)
3: Health				
	**Trauma/injuries (8)**			
	Superficial wounds	8.2 (4)		
	Penetrating wounds	2.0 (1)		
	**Skin illness/disease (8)**			
	Present	2.0 (1)		
	**Respiration (33)**			
	Unusual respiratory character	8.2 (4)		
	**Heart rate (3)**			
	**Bleeding/fluid/mucus from orifice (47)**			
	Mucus from mouth	4.1 (2)		
	Mucus from blowhole	4.1 (2)		
	Dark-green fluid from anus	6.1 (3)		
4: Behavioural interactions				
	**Curvature of peduncle (49)**		**Pod members (49)**	
	Left	12.2 (6)	Present	95.9 (47)
	Right	8.2 (4)	**Status pod members (49)**	
			All alive	4.1 (2)
			All dead	2.0 (1)
			Alive and dead	89.8 (44)
			Stranded	93.9 (46)

Each indicator assessed per domain is boldened.

**Table 5:** Observed prevalence (% of individuals displaying behaviour), mean frequency (rate/minute) ± SEM of point behaviours, and mean relative duration (% of monitored time and range) of state behaviours for only long-finned pilot whales that showed the behaviour, from a total of 31 dry- and 18 in-water-stranded individuals across 11 stranding events on the New Zealand coast between 2010 and March 2022. ^†^ Only prevalent indicators could be tested for statistical differences; * significant difference (*α* = 0.05) in prevalence between stranding circumstances.

	Prevalence		Frequency		Relative Duration	
Behaviour	Dry	In-Water	Dry	In-Water	Dry	In-Water
**State behaviours**						
Ventral recumbency	90.3	94.4			97.7 (81.9–100.0)	73.3 (7.7–99.9)
Lateral recumbency	16.1	50.0			63.7 (4.4–99.8)	59.1 (7.2–97.3)
Dorsal recumbency	0.0	5.6			0.0 (0.0–0.0)	3.4 (3.4–3.4)
Tail flutter ^†^	67.7	72.2			58.1 (4.6–99.9)	49.0 (13.0–92.4)
Dorsal fin flutter ^†^	38.7 *	83.3 *			36.6 (6.1–87.6)	45.2 (12.3–97.3)
Head lift ^†^	38.7 *	72.2 *			12.7 (2.6–32.6)	11.9 (2.3–32.4)
Tail lift ^†^	29.0 *	77.8 *			22.2 (0.6–60.3)	24.9 (2.3–72.9)
Head side-to-side ^†^	29.0 *	66.7 *			25.7 (5.5–81.5)	19.5 (1.1–47.4)
Pec fin flutter R	25.8	22.2			61.2 (3.8–98.6)	50.8 (30.0–93.7)
Pec fin flutter L	12.9	38.9			29.4 (4.3–69.2)	37.8 (7.0–87.0)
Pec joint moves	19.4	22.2			18.3 (1.2–46.2)	28.9 (0.5–78.3)
Tail hover	16.1	22.2			26.1 (2.2–55.2)	16.9 (0.2–38.7)
Tail side-to-side	0.0	44.4			0.0 (0.0–0.0)	15.8 (0.3–46.3)
Body tremble	9.7	16.7			28.3 (0.2–84.2)	20.3 (8.6–27.6)
Vocalisation	3.2	22.2			7.0 (7.0–7.0)	24.1 (5.1–60.2)
Body rocking	16.1	0.0			10.4 (5.2–23.2)	0.0 (0.0–0.0)
Eye open L	6.5	16.7			44.0 (14.2–73.7)	29.7 (2.4–61.9)
Eye open R	6.5	11.1			10.9 (2.5–19.3)	33.4 (2.9–63.8)
Body tenses	3.2	11.1			8.4 (8.4–8.4)	12.5 (5.1–20.0)
Tail arch	0.0	11.1			0.0 (0.0–0.0)	15.8 (12.4–19.1)
Tail fluke slapping	6.5	0.0			28.3 (19.9–36.7)	0.0 (0.0–0.0)
Whole body arching/thrashing	0.0	11.1			0.0 (0.0–0.0)	5.4 (2.4–8.4)
Mouth open	0.0	5.6			0.0 (0.0–0.0)	17.2 (17.2–17.2)
**Point behaviours**						
Blowhole twitch	16.1	38.9	5.6 ± 2.9	6.1 ± 2.3		
Nuchal pad twitch	6.5	11.1	9.5 ± 7.6	5.8 ± 2.1		
Open and close blowhole	3.2	16.7	0.4 ± 0.0	3.1 ± 1.6		
Water from blowhole	3.2	5.6	3.6 ± 0.0	0.7 ± 0.0		
Head–pec fin jerk/flinch	0.0	16.7	0.0 ± 0.0	1.4 ± 0.7		
Movement in lower jaw	0.0	5.6	0.0 ± 0.0	6.9 ± 0.0		
**Physiological parameters**						
Respiration rate ^†^	67.7	61.1	4.1 ± 0.6	4.9 ± 0.8		
Heartbeat	9.7	0.0	48.8 ± 11.6			

**Table 6:** Types of human intervention that occurred with individual focal stranded pilot whales. Prevalence (% of individual focal stranded cetaceans that the intervention occurred with) and relative duration (% and range) of human intervention with individual focal stranded pilot whales (*n* = 49) calculated for those individuals undergoing the intervention type across 11 stranding events between 2010 and March 2022. See Table S3 for descriptions of intervention types.

Intervention	Prevalence	Relative Duration of Individual Monitoring
Present	100.0	97.4 (35.5–100)
Watering	65.3	36.0 (0.5–86.8)
Touching	59.2	61.1 (3.3–100)
Digging	36.7	51.3 (4.6–99.8)
Rolling	24.5	33.8 (0.4–93.5)
Noise	8.2	61.2 (16.4–98.8)
Holds dorsal fin	6.1	35.0 (2.9–97.6)
Places sand by sides	2.0	96.8 (96.8–96.8)
Rubbing	2.0	21.6 (21.6–21.6)

## Data Availability

Restrictions apply to the availability of these data. Data were obtained from the Department of Conservation and members of the public and are available from the authors with the permission of mana whenua, the Department of Conservation, and the public that provided the video data.

## Supplementary Materials

The following supporting information can be downloaded at: https://www.mdpi.com/article/10.3390/ani12141861/s1. **Figure S1**: Observation of dark green liquid (within black ovals) defecated from live-stranded long-finned pilot whale. Photo credit: Rob Leenheer; Figure S2: Observation of pectoral fin oriented laterally and superior to dorsal plane (within black ovals) in live stranded long-finned pilot whale. Photo credit: Kyle Mulinder; Table S1: Stranding events (*n* = 14) and details of video footage collected of individual live cetaceans (*n* = 53) of four odontocete species between August 2010 and March 2022, New Zealand. Only pilot whale data were used in analyses, with data from other species providing ground-truthing to identified behavioural indicators. In the case of mass strandings, footage may have included multiple individuals, however the video length noted included only the focal animal. *Note three animals were filmed both cranio-laterally and laterally; Table S2: Ethogram of stranded odontocete behaviour derived from video observations of 53 focal individuals (4 species, 14 stranding events) on the New Zealand coast between August 2010 and March 2022. Two physiological parameters are included. Note: behaviours displayed only by pilot whales** vs those not displayed by pilot whales*; Table S3: Types of human intervention that occurred with individual focal stranded cetaceans (*n* = 53; 4 species across 14 stranding events) on the New Zealand coast between August 2010 and March 2022.
