# Supplementary material for: Evaluating Potential Cetacean Welfare Indicators from Video of Live Stranded Long-Finned Pilot Whales (Globicephala melas edwardii)
Source: Animals (Basel). 2022 Jul 21;12(14):1861. doi: 10.3390/ani12141861 (PMC9312325; doi:10.3390/ani12141861)
Supplement: Supplementary file 1 [file animals-12-01861-s001.zip › animals-1803463-supplementary.pdf]

Supplementary Materials

Evaluating potential cetacean welfare indicators from video of live stranded long-finned pilot whales (*Globicephala melas edwardii*)

Table S1. Stranding events (n=14) and details of video footage collected of individual live cetaceans (n=53) of four odontocete species between August 2010 and March 2022, New Zealand. Only pilot whale data were used in analyses, with data from other species providing ground-truthing to identified behavioural indicators. In the case of mass strandings, footage may have included multiple individuals, however the video length noted included only the focal animal. *Note three animals were filmed both cranio-laterally and laterally.

Year	Location	Species	Stranding type	No. animals filmed	Video length (mins)	Source	Filming equipment	No. filmed cranio-laterally	No. filmed laterally
2010	Northland	Long-finned pilot whale	Mass	1	0.5	Public	Video camera	1	0
2011	Golden Bay	Long-finned pilot whale	Mass	2	0.8	Public	Phone	0	2
2014	Golden Bay	Long-finned pilot whale	Mass	1	0.3	Public	Phone	1	0
2015	Golden Bay	Long-finned pilot whale	Mass	20	11.3	Public	GoPro	4	16
2017	Golden Bay	Long-finned pilot whale	Mass	5	1.2	Public	GoPro	4	1
2020	Waipu	Pygmy killer whale	Mass	2	294.4	Researcher	GoPro	2	0
2020	Whanganui	Long-finned pilot whale	Single	1	37.4	DOC	Phone	1*	1*
2020	Coromandel	Long-finned pilot whale	Mass	2	2.9	Public	Phone	0	2
2020	Raglan	Cuvier's beaked whale	Single	1	33.1	DOC	Phone	1	0
2021	Ruakaka	Grey's beaked whale	Mass	1	6.2	Public	Phone	0	1
2021	Golden Bay	Long-finned pilot whale	Mass	11	5.9	Public	GoPro	4	7
2021	Taranaki	Long-finned pilot whale	Mass	1	15.4	Public	Phone	1*	1*
2021	Christchurch	Long-finned pilot whale	Single	1	4.1	DOC	Phone	1*	1*
2022	Golden Bay	Long-finned pilot whale	Mass	4	13.8	Public	Phone	4	0
**Total**				**53**	**427.2**			**24***	**32***

Table S2. Ethogram of stranded odontocete behaviour derived from video observations of 53 focal individuals (4 species, 14 stranding events) on the New Zealand coast between August 2010 and March 2022. Two physiological parameters are included. Note: behaviours displayed only by pilot whales** vs those not displayed by pilot whales*.

Body location	Type	Behaviour	Description
Blowhole	Point	Blowhole twitch	Blowhole makes small muscular twitching movement
Blowhole	Point	Water from blowhole	Small amount of water expelled from blowhole without respiration
Blowhole	Point	Open-close blowhole**	Blowhole opens and closes but there is no movement of thorax indicating that respiration does not occur
Fin	State	Dorsal fin flutter	Dorsal fin makes small lateral shaking/tremor type movements
Fin	State	Pec fin flutter L	Pectoral fin left flutters in small shaking/tremor type movements dorso-ventrally
Fin	State	Pec fin flutter R	Pectoral fin right flutters in small shaking/tremor type movements dorso-ventrally
Fin	State	Tail flutter	Tail fluke flutters, small shaking/tremor type movements dorso-ventrally without lifting peduncle
Fin	State	Pec joint moves**	Joint of pectoral fin rotates anterior or posterior without lifting pectoral fin
Head	State	Head arch*	Head and thorax are lifted high off the ground into a curved arch
Head	State	Head lift	Head is lifted slightly off the ground without moving the thorax
Head	State	Head side-to-side	Head moves from side to side laterally
Head	State	Mouth open	Animal opens its mouth and closes it again
Head	Point	Movement in lower jaw	Small twitching type muscular movement in lower jaw/throat as if swallowing
Head	Point	Nuchal pad twitch**	Muscle twitch in nuchal fat pad behind the blowhole
Head	Point	Head-pec fin jerk/flinch**	Anterior part of body from pectoral fin to the head jerks/flinches suddenly to one side
Head	State	Eye open L**	Left eye is open continuously
Head	State	Eye open R**	Right eye is open continuously
Melon	State	Vocalisation**	Animal audibly vocalises

Posture	State	Dorsal recumbency**	Animal is lying on its dorsal (back) surface
Posture	State	Lateral recumbency	Animal is lying on its side (lateral)
Posture	State	Ventral recumbency	Animal is lying on its ventrum (underside)
Tail	State	Tail arch	Tail fluke and entire peduncle are lifted high off the ground into a curved arch
Tail	State	Tail hover	Tail is lifted slightly off ground and remains there hovering
Tail	State	Tail lift	Tail fluke and caudal peduncle lift slightly off the ground
Tail	State	Tail side-to-side	Tail moves from side to side (lateral)
Tail	State	Tail fluke slapping**	Animal slaps fluke up and down (dorso-ventral) on ground quickly and vigorously without lifting peduncle
Whole body	State	Body rocking	Entire body rocks laterally side to side
Whole body	State	Body tenses	Entire body girth appears to expand without respiration, possibly tensing all muscles
Whole body	State	Body tremble	Whole body trembles/shakes
Whole body	State	Whole body arching/ thrashing	Entire body thrashes/arches vigorously dorso-ventrally
Thorax	Point	Heartbeat**	Visible heartbeat close to left pectoral fin insertion on the ventrum
Thorax	Point	Respiration	Animal’s thorax expands, blowhole opens, and audible explosive exhalation and inhalation occurs

Table S3. Types of human intervention that occurred with individual focal stranded cetaceans (n=53; 4 species across 14 stranding events) on the New Zealand coast between August 2010 and March 2022.

**Intervention**	**Description of intervention**
Present	Human within ~2m of individual focal stranded cetacean but not touching it
Watering	Human pours water onto cetacean
Touching	Human places hands on cetacean but is not trying to move it
Digging	Human digs out sand from around cetacean but has no direct contact
Rolling	Human rolls cetacean laterally (direct contact always occurs with rolling)
Noise	Noise caused by humans (other than normal talking) within ~2m of individual focal stranded cetacean
Holds dorsal fin	Human holds onto dorsal fin of cetacean using hands
Dog present	Canine is within ~2m of individual focal stranded cetacean but has no direct contact
Reflex test	Human performs reflex tests (palpebral, eyes or blowhole) on cetacean
Places block by sides	Human places wooden blocks or similar each lateral side of cetacean to prevent movement
Places sand by sides	Human builds sand wall at lateral sides of cetacean to prevent rolling
Holds peduncle	Human encircles peduncle of cetacean using arm
Rubbing	Human uses cloth to rub skin of focal stranded cetacean

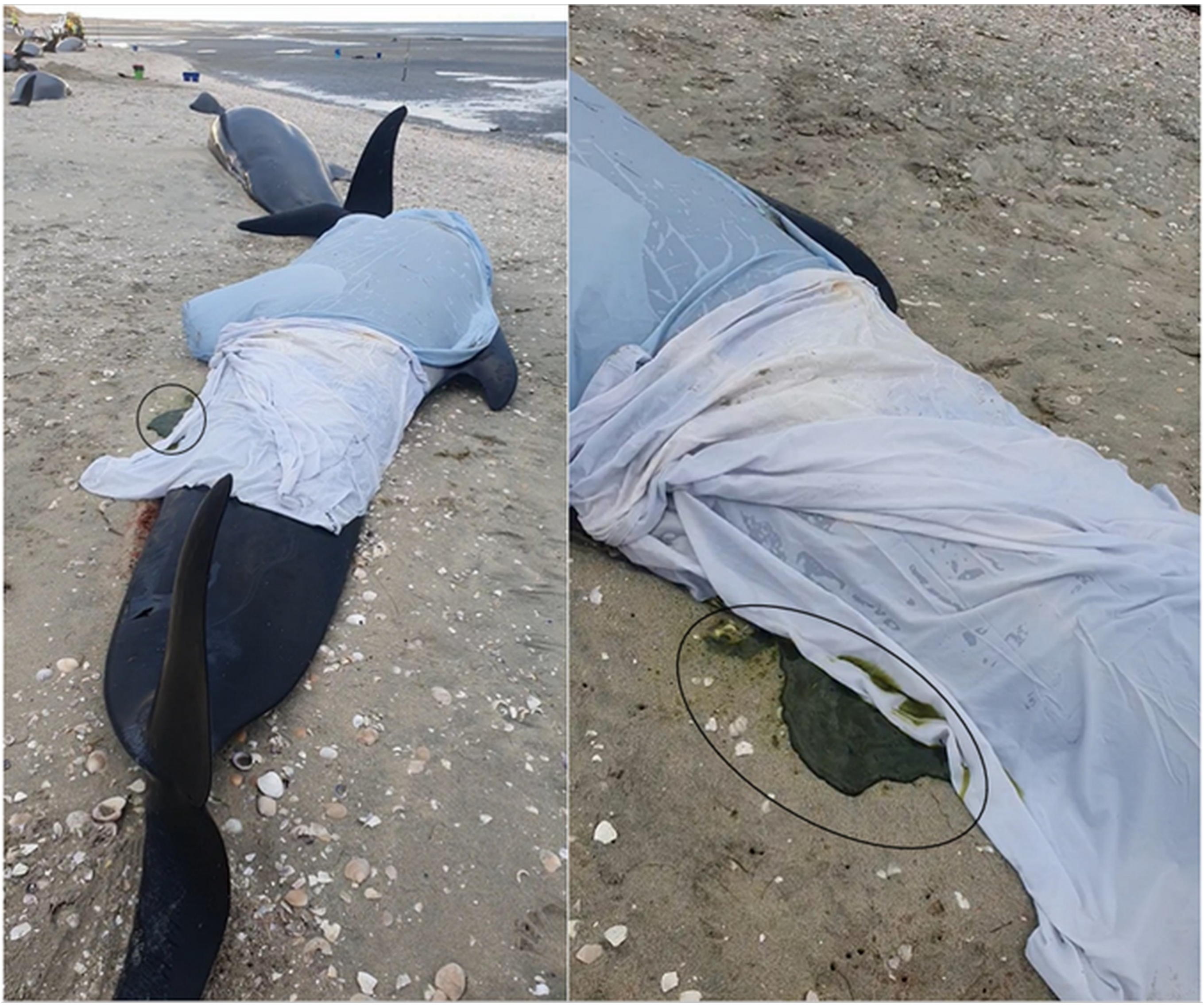

Figure S1. Observation of dark green liquid (within black ovals) defecated from live-stranded long-finned pilot whale. Photo credit: Rob Leenheer.

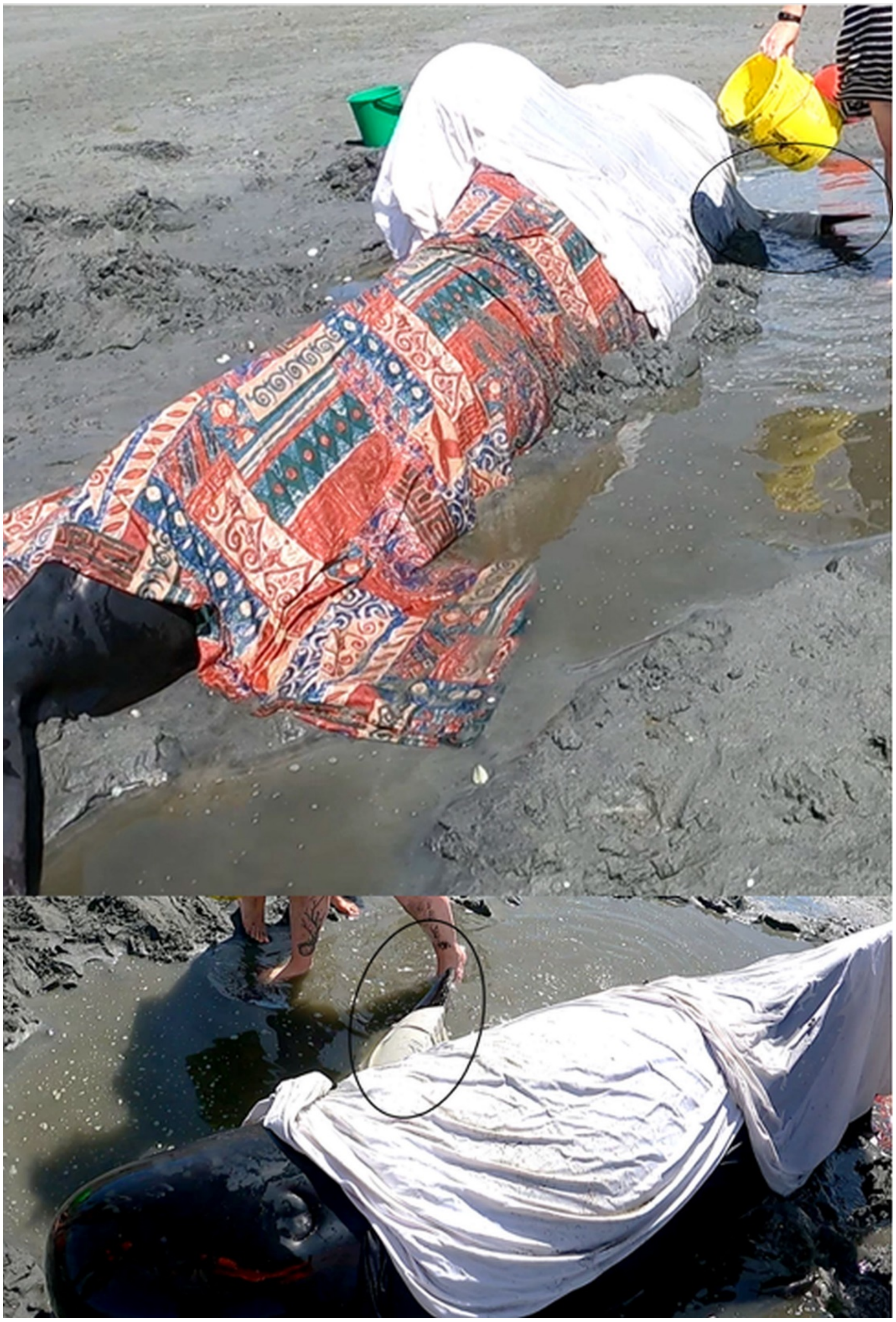

Figure S2. Observation of pectoral fin oriented laterally and superior to dorsal plane (within black ovals) in live stranded long-finned pilot whale. Photo credit: Kyle Mulinder.
